# Predictors of Symptom Duration and Bacteriuria in Uncomplicated Urinary Tract Infection

**DOI:** 10.1080/02813432.2018.1499602

**Published:** 2018-09-03

**Authors:** Marianne Bollestad, Ingvild Vik, Nils Grude, Morten Lindbæk

**Affiliations:** a The Antibiotic Centre of Primary Care, Department of General Practice, Institute of Health and Society, University of Oslo, Oslo, Norway;; b Oslo Accident and Emergency Outpatient Clinic, Department of Emergency General Practice, City of Oslo Health Agency, Oslo, Norway;; c Division of Medicine, Stavanger University Hospital, Stavanger, Norway;; d Department of Medical Microbiology, Vestfold hospital trust, Toensberg, Norway

**Keywords:** Primary health care, urinary tract infection, after-hours care, bacteriuria, female urogenital diseases

## Abstract

**Objective:** To identify baseline predictors of symptom duration after empirical treatment for uncomplicated urinary tract infection (UTI) and significant bacteriuria in a cohort of women treated for UTI.

**Design:** Prospective single-centre cohort study.

**Setting:** Outpatient clinic in Norway.

**Patients:** From September 2010 to November 2011, 441 women aged 16–55 years with symptoms of uncomplicated UTI were included.

**Results:** Dipstick findings of leukocyte esterase 1 + (incidence rate ratio (IRR) 1.93, 95% confidence interval (CI) 1.23–3.01, *p* < 0.01) and microbe resistant to mecillinam treatment (IRR 1.41, 95% CI 1.07–1.89, *p* = 0.02) predicted longer symptom duration. More pronounced symptoms did not predict longer symptom duration (IRR 1.18, 95% CI 0.94–1.46, *p* = 0.15) or significant bacteriuria (odds ratio [OR] 1.16, 95% CI 0.72–1.88, *p* = 0.54). Leukocyte esterase 2 + (OR 2.51, 95% CI 0.92–6.83, *p* = 0.07) or 3 + (OR 2.40, 95% CI 0.88–6.05, *p* = 0.09) and nitrite positive urine dipstick test (OR 3.22, 95% CI 1.58–7.01, *p* = <0.01) were associated with bacteriuria.

**Conclusion:** More pronounced symptoms did not correlate with significant bacteriuria or symptom duration after empirical treatment for acute cystitis. One might reconsider the current practice of treating uncomplicated UTI based on symptoms alone.

**Key Points**
- Treatment strategies for milder infectious diseases must consider ways of reducing antibiotic consumption to decelerate the increase in antibiotic resistance. Our findings suggest that more emphasis should be put on urine dipstick results and bacteriological findings in the clinical setting. One might reconsider the current practice of treating uncomplicated UTIs based on symptoms alone.

## Introduction

Urinary tract infections (UTIs) are one of the most common bacterial infections encountered in the primary care setting. Most women experience at least one episode of acute uncomplicated cystitis in their lifespan [1, 2]. Women report diversity in symptom burden in relation to an episode of uncomplicated UTI [3].

Antibiotics prescribed in primary care account for around 85% of the total human antibiotic consumption in Norway [4]. Empirical treatment of UTIs is based on updated knowledge of antimicrobial resistance and national guidelines [5,6]. Refraining from treatment has been found to be inferior for both symptomatic and bacteriological cure of uncomplicated UTI [7–9]. However, a study of ibuprofen versus fosfomycin for the treatment of uncomplicated UTI found that two-thirds of women recovered without antibiotics [10]. Subgroup analysis of the patients found that five factors predicted the need for subsequent antibiotic treatment: urgency/frequency, impaired daily activities, and positive urine dipstick test results for erythrocytes, leukocyte esterase and nitrite [11].

Both national and international guidelines endorse the treatment of acute uncomplicated cystitis based on symptoms alone [5,12]. Studies have found diverging results in terms of how well symptoms predict the presence of a UTI using a positive urine culture for known uropathogens as the gold standard [7,13–15]. Women with a history of cystitis, frequent somatic symptoms, presence of bacteria resistant to the chosen antibiotic regime and severe symptoms at baseline are more likely to have symptoms of acute cystitis lasting longer than three days after the initiation of treatment [16].

Detection of leukocyte esterase and nitrites or erythrocytes and nitrites on the urine dipstick is considered to be moderately sensitive and specific for detecting a UTI when using a positive culture as the gold standard [14,17,18]. One study showed that a negative urine dipstick test for leukocytes and nitrites accurately predicted the absence of infection, as defined by standard microbiological parameters, but it did not predict the response to antibiotic treatment. Antibiotic treatment significantly reduces dysuria in women with a negative urine dipstick test [19].

Antibiotic resistance rates are increasing, and medical practice now focuses on developing new treatment strategies to reduce antibiotic use without comprising patient safety. New generations of drugs to combat microbial infections will probably not be a reality in the foreseeable future, and clinicians need alternative strategies to reduce antibiotic consumption [20].

It is of interest to identify which clinical and bacteriological factors, if any, predict longer duration of symptoms following empirical antibiotic treatment and the presence of bacteriuria. Further studies are needed to develop good clinical decision aids to identify those patients who are suitable for symptomatic treatment and/or delayed prescribing for uncomplicated UTI. This could lead to decreased antibiotic consumption and at the same time minimize the potential for prolonged patient discomfort.

The aims of this study were to identify:

- factors that predict significantly longer duration of symptoms in patients with uncomplicated UTI after empirical antibiotic treatment
- factors that predict significant bacteriuria in patients presenting with an uncomplicated UTI.

## Material and methods

**Figure 1.**
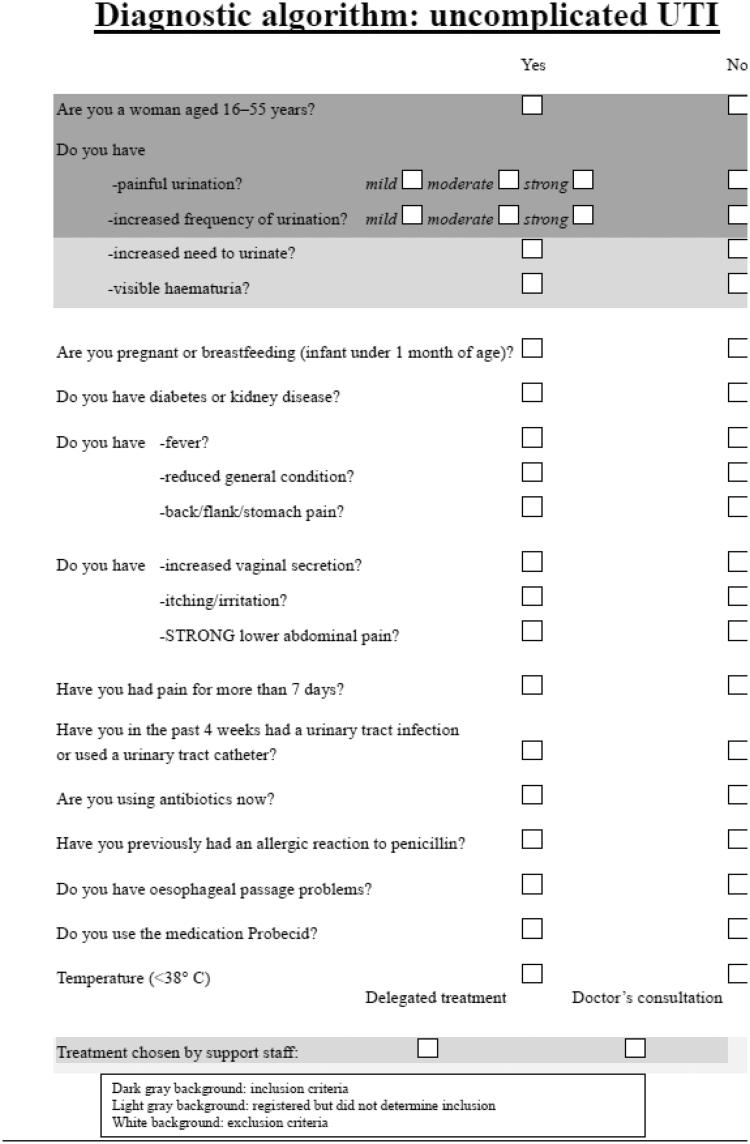
Data were collected at Oslo Accident and Emergency Outpatient Clinic, Norway 2010–2011. The results have been previously published: http://www.tandfonline.com/doi/full/10.3109/02813432.2015.1041827 [20].

The analysed data were based on findings from a study assessing the use of a diagnostic algorithm to identify patients with uncomplicated UTI [21]. A prospective randomized study was performed during the 14 months from September 2010 to November 2011. Women between the ages of 16 and 55 years presenting with dysuria and increased frequency of urination were included. Visible haematuria and increased urinary urgency were also registered but were not used to determine inclusion. The exclusion criteria were significant co-morbidity including diabetes or kidney disease, pregnancy, breastfeeding (infant under one month of age), symptoms of pyelonephritis, symptoms of a sexually transmitted disease, symptoms lasting longer than seven days, use of a urinary catheter or diagnosed a urinary tract infection in the last four weeks, current use of antibiotics, known allergy to penicillin, oesophageal passage problems, use of the medication probenecid and fever (>38°C) (Figure 1). The patients enrolled were randomized into two groups by drawing numbers from an opaque envelope. One group received treatment with mecillinam according to the standard diagnostic algorithm, and the control group was seen by a doctor who was unaware that the patient was identified as eligible for treatment according to the diagnostic algorithm.

The cardinal symptoms of acute uncomplicated UTI (painful urination and increased frequency of urination) were registered as mild, moderate or severe upon presentation to the study nurse.

The follow-up included a telephone call from the study co-ordinator one week after the primary contact and two weeks after the treatment was completed. The number of days until symptom resolution was recorded. Patients without symptom resolution at the two-week follow-up were registered as symptomatic for 15 days for the purpose of statistical analysis. Urine dipstick findings on the day of presentation were recorded. The Multistix 5 dipstick produced by Siemens Healthcare Diagnostics AS was used. The urine dipstick results were graded from 0 to 4 for erythrocytes, 0–3 for leukocyte esterase, and positive/negative for nitrite. A urine sample was sent to the laboratory for culturing on the day of presentation and one week after completing the treatment. The women were instructed to collect a midstream urine sample after spreading of the labia. The initial sample was taken upon presentation, regardless of bladder incubation time. For the follow-up sample the included patients were instructed to send a morning urine sample. The vast majority of samples were received within one to two weeks after treatment.

During the inclusion period, 832 consecutive women were screened for eligibility to enter the study; 391 were excluded primarily because they presented with one or more exclusion criteria. A total of 441 women were randomized to receive treatment in accordance with the diagnostic algorithm (*n* = 245) or treatment following a regular doctor’s consultation (*n* = 196). A total of eight patients were excluded due to deviations from protocol leaving 242 in the diagnostic algorithm group versus 191 who received treatment following a doctor’s consultation. The trial flow chart is included as a supplementary file. The cohort represents a young population with a mean age of 27 years [21].

All urine samples were cultured according to established procedures for identification of microbes with resistance patterns at the Department of Medical Microbiology, Oslo University Hospital, Ullevål. The uropathogens were quantified in colony-forming units (cfu)/mL. Significant bacteriuria was defined according to current European guidelines for patients with symptoms of UTI as ≥10^3^ cfu/mL for primary pathogens, ≥10^4^ cfu/mL for secondary pathogens and ≥10^5^ cfu/mL for doubtful pathogens [22]. Bacterial growth which did not meet the criteria for significant bacteriuria was registered as a negative culture.

Most patients included in this study were treated with a three-day regimen of mecillinam, dosage 200 mg TID for three days. Other treatment regimens were trimethoprim and nitrofurantoin, treatment length varied from three to 7 days.

The urine from 57% of patients exhibited significant bacterial growth. The most common pathogens were *Escherichia coli (E. coli)* (77%) and *Staphylococcus saprophyticus (S. saprophyticus)* (16%) [21].

The number of days with symptoms of acute UTI before the patient sought health care was not registered, but it was less than seven days for all patients since longer duration of symptoms led to exclusion. A median of three days until symptom resolution was found in both groups (*p* = 0.3) [21].

### Statistical methods

To identify factors predicting longer symptom duration, the traditional negative binomial (NB2) model was fitted to the count data. The NB2 model was selected to handle over-dispersed Poisson data. Over-dispersion of the data was confirmed with the Pearson dispersion statistic of 4.31, a value that exceeds 1 for equally dispersed Poisson data.

Selection of predictors into the final fitted model was based on the backward elimination method. We started with all possible predictors of longer system duration in the model and at each stage of model development, the predictor with the largest *p*-value was removed and the model refitted. We also estimated the Akaike Information Criterion (AIC), which we used to compare all subsequent models; the smaller the AIC, the better the model. Therefore, each subsequent step of modelling eliminated the least significant variable in the model until the AIC estimate was higher than in the previous step. However, at each stage of modelling, the variable age was retained in the model regardless of its predictive power.

For the purpose of analysis, the cohort was divided into three age groups: 16–22, 23–28, and ≥29 years.

To identify factors predicting significant bacteriuria sensitivity, specificity and positive predictive values (PPV) were first calculated.

Binary logistic regression models were also fitted to the data to identify predictors of significant bacteriuria. The analyses proceeded in two steps. Firstly, univariate logistic regression models were fitted to the data to identify significant predictors. Secondly, the significant predictors from the univariate analyses together with clinically relevant predictors were used to fit a multivariate logistic regression model.

To assess the accuracy and discriminative value of the final prediction model, sensitivity was plotted against the false-positive rate (1–specificity) over a range of cut-point values for the continuous linear score in the receiver-operating characteristic (ROC) space, and the area under the ROC curve (AUC) was calculated.

All statistical analyses were performed using SPSS 22, and significance was set at *p* < 0.05.

## Results

More severe symptoms or the presence of significant bacteriuria were not significant predictors of longer symptom duration after empirical treatment. The presence of leukocyte esterase 1+ on the urine test strip and the significant growth of a microbe resistant to the given antibiotic predicted significantly longer symptom duration (Table 1).

**Table 1.** Predictors of a longer duration of urinary tract symptoms after empirical antibiotic treatment.

	Univariate analyses			Multivariate analyses		
	IRR* (B)	95% CI	*p*	IRR (B)	95% CI	*p*
Two strong symptoms and urgency *n* = 89	1.12	0.92–1.37	0.27	1.18	0.94–1.46	0.15
(reference: weak symptoms *n* = 344)						
Age, years						
(reference: 16–22)						
16–22 *n* = 151	1.0			1.0		
23–28 *n* = 146	1.05	0.87–1.28	0.60	1.04	0.83–1.29	0.76
≥29 *n* = 136	1.14	0.94–1.39	0.19	1.06	0.85–1.32	0.59
Leukocytes						
(reference: negative *n* = 44)						
3+ *n* = 121	0.82	0.61–1.10	0.19	1.22	0.80–1.86	0.37
2+ *n* = 129	0.86	0.64–1.14	0.30	1.18	0.78–1.80	0.43
1+ *n* = 55	1.27	0.91–1.75	0.16	1.93	1.23–3.01	<0.01
Leukocytes, all positive *n* = 305	0.92	0.70–1.20	0.53			
(reference: negative *n* = 44)						
Nitrite positive *n* = 58	1.13	0.89–1.44	0.31			
(reference: negative *n* = 289)						
Erythrocytes positive *n* = 294	0.82	0.64–1.05	0.11			
(reference: negative *n* = 54)						
Negative urine dipstick *n* = 22	0.86	0.60–1.23	0.41	0.65	0.38-1.12	0.12
(reference: any positive dipstick finding *n* = 324)						
Microbe susceptibility to given treatment						
(reference: sensitive *n* = 179)						
Resistant *n* = 47	1.51	1.16–1.97	<0.01	1.41	1.07–1.89	0.02
No susceptibility testing performed *n* = 187	1.02	0.85–1.21	0.86	0.92	0.75–1.12	0.40
Doctors’ consultation *n* = 191	1.12	0.95–1.31	0.18			
(reference: algorithm group *n* = 242)						
Significant bacteriuria *n* = 233	1.06	0.89–1.25	0.53			
(reference: negative *n* = 173)						

* IRR = incidence rate ratio;

CI = confidence interval.

The predictors of longer duration of urinary tract symptoms after empirical antibiotic treatment were identified using the traditional negative binomial model. The data were collected at Oslo Accident and Emergency Outpatient Clinic, Norway, 2010–2011.

The presence of nitrite on the urine dipstick gave a PPV of 0.81 for significant bacteriuria; this was the highest PPV found. The presence of leukocyte esterase of 2+ and 3+ was also associated with an increased PPV of significant bacteriuria but to a lesser degree. More severe symptoms did not predict significant bacteriuria (Table 2).

**Table 2.** Symptoms and signs that predicted significant bacteriuria in acute uncomplicated cystitis.

Symptoms and signs	Growth + (%)	Growth – (%)	Total (%)	Sensitivity	Specificity	PPV*
	*N* = 233	*N* = 173	*N* = 406			
Pain at urination (all)	233 (100)	173 (100)	406 (100)			
Strong	77 (33)	54 (31)	131 (32)	0.33	0.69	0.58
Urinary frequency (all)	233 (100)	173 (100)	406 (100)			
Strong	81 (35)	65 (38)	146 (36)	0.35	0.62	0.56
Urgency (*n* = 405)^^^	228 (98)	172 (99)	400 (99)	0.98	0.58	0.57
Two strong symptoms and urgency	53 (23)	35 (20)	88 (22)	0.23	0.80	0.60
Age, years (all)	233 (100)	173 (100)	406 (100)			
16–22	73 (31)	67 (39)	140 (35)	0.31	0.61	0.52
23–28	80 (34)	57 (33)	137 (34)	0.34	0.67	0.58
≥29	80 (34)	49 (28)	129 (32)	0.34	0.72	0.62
Urine dipstick findings						
Leukocytes (*n* = 327)	192 (59)	135 (41)				
3+	75 (39)	40 (30)	115 (32)	0.39	0.70	0.65
2+	79 (41)	39 (29)	118 (36)	0.41	0.71	0.67
1+	25 (13)	27 (20)	52 (16)	0.13	0.80	0.48
Leukocytes, all positive	179 (93)	106 (79)	285 (87)	0.93	0.21	0.63
Negative	13 (7)	29 (22)	42 (13)			
Nitrite positive (*n* = 325)	44 (23)	10 (8)	54 (17)	0.23	0.93	0.81
Erythrocytes positive (*n* = 326)	174 (91)	103 (77)	277 (85)	0.91	0.23	0.63

* PPV = positive predictive value.

The sensitivity, specificity and PPV of symptoms and signs that predicted significant bacteriuria were identified using data collected at Oslo Accident and Emergency Outpatient Clinic, Norway, 2010–2011.

^ 1 patient did not respond.

Logistic regression analysis showed that neither age nor more pronounced clinical symptoms upon presentation correlated with the presence of significant bacteriuria.

Urine dipstick positive for nitrite (OR 3.22, 95% CI 1.58–7.01, *p* < 0.01) was associated with an increased probability of significant bacteriuria (Table 3). Urine dipstick findings with a leukocyte esterase value of 3 + (OR 2.40, 95% CI 0.88–6.05, *p* = 0.09), or a leukocyte esterase value of 2 + (OR 2.51, 95% CI 0.92–6.83, *p* = 0.07) showed association with significant bacteriuria, but did not reach significance.

**Table 3.** Predictors of the presence of significant bacteriuria in urine samples from women with diagnosed acute uncomplicated cystitis.

	Univariate analyses			Multivariate analyses		
	OR* (B)	95% CI	*p*	OR (B)	95% CI	*p*
Two strong symptoms and urgency, *n* = 89	1.16	0.72–1.88	0.54			
(reference: weak, *n* = 344)						
Age, years						
16–22, *n* = 151	1.00			1.00		
(reference)						
23–28, *n* = 146	1.29	0.80–2.07	0.30	1.35	0.75–2.40	0.31
≥29, *n* = 136	1.50	0.92–2.44	0.10	1.44	0.80–2.56	0.22
Urine dipstick findings						
Leukocytes	1.0			1.0		
(reference: negative, *n* = 44)						
3+, *n* = 121	4.18	1.96–8.93	<0.01	2.40	0.88–6.05	0.09
2+, *n* = 129	4.52	2.12–9.65	<0.01	2.51	0.92–6.83	0.07
1+, *n* = 55	2.07	0.88–4.84	0.10	1.38	0.46–4.19	0.57
Leukocytes, all positive, *n* = 305	3.77	1.88–7.56	<0.01			
(reference: negative, *n* = 44)						
Nitrite positive, *n* = 58	3.71	1.79–7.68	<0.01	3.22	1.58–7.01	<0.01
(reference: negative, *n* = 289)						
Erythrocytes positive, *n* = 294	2.91	1.55–5.46	<0.01	1.40	0.60–3.25	0.45
(reference: negative, *n* = 54)						
Positive urine dipstick, 324 (reference: negative, *n* = 22)	9.18	2.63–32.02	<0.01	2.73	0.47–15.73	0.26

* OR = odds ratio;

CI = confidence interval.

The predictors of the presence of significant bacteriuria were identified in a logistic regression analysis of data collected at Oslo Accident and Emergency Outpatient Clinic, Norway, 2010–2011.

**Figure 2.**
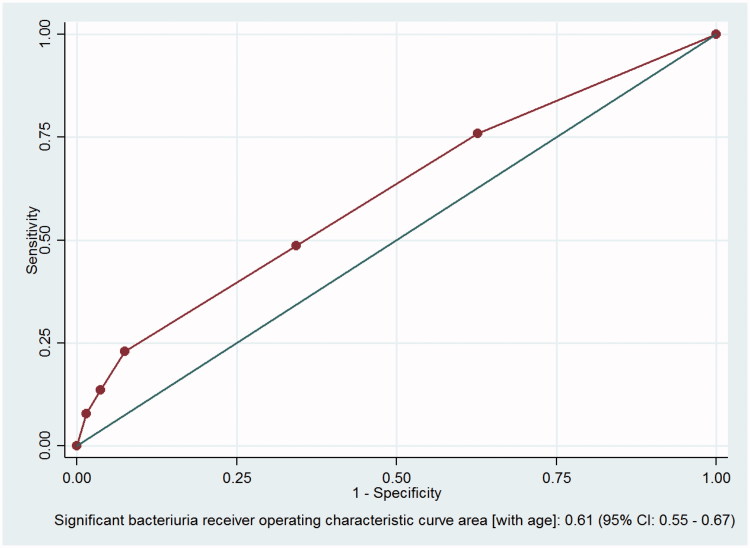
Correlations between the presence of leukocyte esterase, nitrite, age and significant bacteriuria. CI = confidence interval. Receiver-operator characteristic (ROC) analysis of the relationship between the presence of leukocyte esterase, nitrite, age and significant bacteriuria. The data were collected at Oslo Accident and Emergency Outpatient Clinic, Norway, 2010–2011.

The ROC analysis gave an AUC of the final model of 0.61 (95% CI 0.55–0.67) (Figure 2).

In our cohort, 59/347 (17.0%) of the registered urinary dipstick findings were nitrite positive. For five of the patients with a positive nitrite dipstick test, a urine culture was not performed. After exclusion of these patients, 42/54 (77.8%) of nitrite positive urine samples showed significant growth of a gram-negative uropathogen. For the remaining cultures, one showed no growth, two showed significant growth of *S. saprophyticus*, and the rest showed mixed bacterial growth.

We found that 176/180 (97.8%) of *E. coli* isolates were sensitive to mecillinam and 39/146 (26.7%) of the urine samples with significant growth of *E. coli* and registered urinary dipstick findings were nitrite positive.

## Discussion

### Statement of principal findings

Our study identified the presence of leukocyte esterase (2+, 3+) and nitrite on the urinary dipstick test as significant predictors of the presence of bacteriuria. Leukocyte esterase 1+ and microbial resistance to the given antibiotic was associated with longer symptom duration. The severity of symptoms at presentation was neither a significant predictor of the presence of bacteriuria nor longer symptom duration after adequate treatment.

### Strengths and weaknesses of the study

The study included patients recruited from a large and relatively young population, and complete data were obtained from nearly all the included patients. Given the median age of 27 years in these subjects, the findings are representative of younger patient populations compared with those included in previously published studies, and do not represent the elderly population. Women aged ≤55 years represent around 50% of women presenting to the general practitioner with UTIs. The questionnaire did not identify a complete medical history or social and psychological factors that could have affected the reported symptoms. The study did not include a control group of untreated women with symptoms of uncomplicated UTI.

### Findings in relation to other studies

Previous studies have shown varying results in terms of whether symptoms can be used to identify patients with an uncomplicated UTI [8,11,12]. Norwegian national guidelines suggest that antibiotic treatment should be given based on symptoms alone for women with suspected acute uncomplicated UTI [23].

Nitrite-positive urine dipstick was associated with significant bacteriuria. Gram-negative bacteria produce nitrite, and *E. coli* is by far the most common gram-negative uropathogen. A previous study of *E. coli* has identified specific virulence factor genes and phylogenetic groups of the microbe that increase the risk of persistence and relapse of UTI [24].

A relatively low percentage (26.7%) of patients who had significant growth of *E. coli* had a positive nitrite urinary dipstick test. A possible explanation for this finding is that the presence of nitrite on the urinary dipstick is associated with a more virulent isolate and can explain the association with prolonged symptom duration. Negative nitrite findings in the urinary dipstick test are also related to insufficient bladder time for conversion of nitrate to nitrite, decreased urine pH and low urinary excretion of nitrate.

The second most frequently isolated microbe was *S. saprophyticus*, which is considered to be intrinsically resistant to mecillinam treatment. However, a clinical effect has been shown for lower UTI in women where *S. Saprophyticus* as the bacterial agent. It is suspected that the observed effect of mecillinam treatment on microbes intrinsically resistant to treatment is related to the high concentration of mecillinam in urine and relatively low minimal inhibitory concentration of uropathogens [25].

Analysis of factors that might be associated with significant bacteriuria showed that detecting leukocyte esterase 2+ or 3+ on the urine dipstick was more clearly associated with significant bacteriuria than the detection of leukocyte esterase 1+. It is possible that in the cohort where leukocyte esterase 1+ was detected the symptoms did not represent an acute uncomplicated UTI and, if so, the antibiotics would have been expected to be less effective. In addition, the detection of leukocyte esterase 1+ on the urine dipstick was associated with longer symptom duration following empiric antibiotic treatment. We speculate that a possible treatment strategy for this group may be symptomatic relief combined with delayed prescribing.

In this study population, we found significant growth of uropathogens in 57% of the primary urine samples. Previous studies have suggested that for acute uncomplicated UTI, a value ≥10^2^ cfu/mL could signify significant bacterial growth, not ≥10^3^ cfu/mL as defined by current standards [22]. It is possible that cases of clinically relevant bacterial growth are missed and that use of polymerase chain reaction in testing for bacterial agents may improve the diagnostic precision [26].

### Implications

More pronounced symptoms did not correlate with significant bacteriuria or longer symptom duration after empirical treatment for uncomplicated UTI. Our study identified dipstick findings and bacteriological findings as clinically useful for identifying women with an expected longer duration of symptoms after empiric treatment and significant bacteriuria at presentation.

Future treatment strategies for milder infectious diseases must consider ways of reducing antibiotic consumption to decelerate the increase in antibiotic resistance, and several antibiotic-sparing treatment regimens have been proposed [10,27]. Our findings suggest that in clinical practice perhaps more emphasis should be put on urine dipstick results and bacteriological findings. In order to reduce unnecessary use of antibiotics, one might reconsider the current practice of treating uncomplicated UTI based on symptoms alone.
